# A Possible Role of Remdesivir and Plasma Therapy in the Selective Sweep and Emergence of New SARS-CoV-2 Variants

**DOI:** 10.3390/jcm10153276

**Published:** 2021-07-24

**Authors:** Philippe Colson, Christian A. Devaux, Jean-Christophe Lagier, Philippe Gautret, Didier Raoult

**Affiliations:** 1 IHU Méditerranée Infection, 19-21 Boulevard Jean Moulin, 13005 Marseille, France; philippe.colson@univ-amu.fr (P.C.); christian.devaux@mediterranee-infection.com (C.A.D.); jean-christophe.lagier@univ-amu.fr (J.-C.L.); philippe.gautret@ap-hm.fr (P.G.); 2 Microbes Evolution Phylogeny and Infections (MEPHI), Institut de Recherche pour le Développement (IRD), Aix-Marseille University, 27 Boulevard Jean Moulin, 13005 Marseille, France; 3 Assistance Publique-Hôpitaux de Marseille (AP-HM), 264 rue Saint-Pierre, 13005 Marseille, France; 4 CNRS, 13009 Marseille, France; 5 Vecteurs-Infections Tropicales et Méditerranéennes (VITROME), Institut de Recherche pour le Développement (IRD), Aix-Marseille University, 27 Boulevard Jean Moulin, 13005 Marseille, France

**Keywords:** SARS-CoV-2, COVID-19, remdesivir, plasma therapy, anti-spike antibodies, variant, mutants

## Abstract

Since summer 2020, SARS-CoV-2 strains at the origin of the COVID-19 pandemic have suddenly been replaced by new SARS-CoV-2 variants, some of which are highly transmissible and spread at a high rate. These variants include the Marseille-4 lineage (Nextclade 20A.EU2) in Europe, the 20I/501Y.V1 variant first detected in the UK, the 20H/501Y.V2 variant first detected in South Africa, and the 20J/501Y.V3 variant first detected in Brazil. These variants are characterized by multiple mutations in the viral spike protein that is targeted by neutralizing antibodies elicited in response to infection or vaccine immunization. The usual coronavirus mutation rate through genetic drift alone cannot account for such rapid changes. Recent reports of the occurrence of such mutations in immunocompromised patients who received remdesivir and/or convalescent plasma or monoclonal antibodies to treat prolonged SARS-CoV-2 infections led us to hypothesize that experimental therapies that fail to cure the patients from COVID-19 could favor the emergence of immune escape SARS-CoV-2 variants. We review here the data that support this hypothesis and urge physicians and clinical trial promoters to systematically monitor viral mutations by whole-genome sequencing for patients who are administered these treatments.

## 1. Introduction

At the start of the SARS-CoV-2 epidemic in December 2019, a clone appeared called the Wuhan-Hu-1 strain [1], but a mutation (a D614G mutation in the spike protein) was precociously observed in Europe and then carried by the majority of viruses [2]. This mutation seems to have played an important role in the spread of this mutant. In the United States, the observed strains seem to have come from this European mutant and from China [3]. In Europe, as in China, this first strain displayed a typical coronavirus epidemic curve, bell-shaped. In France, the circulation of this first strain stopped in May 2020.

We have had as a strategy, like other countries, to systematically perform SARS-CoV-2 genome sequencing for the purpose of epidemiological surveillance, in particular because RNA viruses present high rates of mutations [4,5,6]. It seems that by the end of this first period of the pandemic in France during late spring 2020, the number of mutations was already undergoing an accelerating increase [7]. In July 2020, an epidemic occurred with a variant we named Marseille-1, for which we have been able to trace an African origin [8]. Then, in August 2020, another epidemic occurred with a different variant we named Marseille-4 (also named Nextstrain 20A.EU2 clade), whose genome comprised 13 mutations compared to the Wuhan-Hu-1 strain and that we were able to link to a mink strain [9]. During this period from May to August 2020, farmed minks were identified as a major source of virus genetic evolution with five clades identified as emerging from mink farms in Holland and Denmark, and it was demonstrated that these strains were transmissible to humans and between humans [10]. SARS-CoV-2 variants could therefore have an epizoonotic source secondary to the human pandemic, which began as a zoonosis [1].

Globally, mutations have appeared on numerous occasions and independently in different viral clades, in particular at positions 501 and 484 of the spike protein, but also at other positions such as positions 452 or 677 [11,12]. This shows that these positions are hot spots of mutations, as they vary commonly and in different genetic backgrounds. Here, we question the role in the emergence of variants of the selective pressure exerted by anti-SARS-CoV-2 spike antibodies occurring naturally as a consequence of a viral infection or induced by a vaccine immunization as well as the possible role of antiviral drugs. We aimed particularly to provide current evidence in favor of the possible role of remdesivir, an inhibitor of the RNA-dependent RNA polymerase (RdRp), and anti-SARS-CoV-2 spike antibodies, alone or in combination, in the genesis of new variants whose epidemic or pathogenic potentials still remain to be deciphered. Patients in whom the emergence of SARS-CoV-2 variants is the most likely are chronic viral carriers [13]. There is no reason that virus evolution differs for such patients from that observed during infections with other RNA viruses such as for instance HIV, for which viral quasi-species harboring antiretroviral drug-resistance mutations rapidly emerge in patients receiving a monotherapy [14]. In the case of SARS-CoV-2 infection, natural evolution with or without treatment is very short, usually in the order of ten to fifteen days, except under special conditions. Thus, viral carriage can be considerably extended among immunocompromised people. The elderly might be at greater risk for prolonged viral shedding due to their weakened immune system. However, viral persistence was significantly associated with older age in some studies [15,16] but not in others [17,18,19]. Extended viral carriage is theoretically prone to the emergence of viral mutants in the case of antiviral therapies, as is observed for HIV infection. Several such patients have received different anti-SARS-CoV-2 treatments that include convalescent plasma from COVID-19 patients, anti-SARS-CoV-2 spike monoclonal antibodies, and/or remdesivir. These treatments failed in a substantial number of cases, indicating that they were not capable of eradicating the virus if the patient was immunodeficient (Table 1).

## 2. Emergence of SARS-CoV-2 Variants Harboring Critical Amino Acid Substitutions and Deletion in the Spike Protein

**Figure 1:**
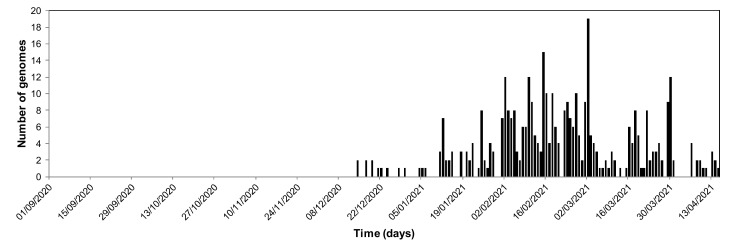
Example of the emergence of the Marseille-501 SARS-CoV-2 spike variant (lineage A.27) in 2020 (number of sequences available from the GISAID database (https://www.gisaid.org/, accessed on 26 May 2021).

Since the start of the COVID-19 pandemic, whole-genome sequencing approaches have made it possible to demonstrate that SARS-CoV-2 variants have gradually replaced the original “Wuhan-Hu-1 strain”. Since July 2020, these variants have caused several overlapping or successive epidemic waves, and some of them have become the majority strains in different geographical areas (Figure 1).

This is, for instance, the case of the variants Marseille-4 [9] (Nextstrain clade 20A.EU2) and Marseille-501 [2] (Pangolin lineage A.27) in Southern France and in Europe [28,29] and of variants 20I/501Y.V1 (lineage B.1.1.7) in the United Kingdom [30,31], 20H/501Y.V2 (lineage B.1.351) in South Africa [32], 20J/501Y.V3 (lineage B.1.281.1) in Brazil [33], and of lineage B.1.617 in India [34]. Compared to the original Wuhan-Hu-1 strain, these variants harbor multiple non-synonymous mutations in their genomes, including some causing amino acid substitutions in the spike glycoprotein (S) that interacts with the ACE2 cellular receptor and is the target of neutralizing antibodies [35,36]. Until autumn 2020, SARS-CoV-2 genetic diversity was most often deemed to be low, as reported in the study of 27,977 SARS-CoV-2 genomes from 84 countries [37] or in analyses suggesting that the diversity in the surface glycoproteins of influenza A viruses was 437-fold greater than that measured in the SARS-CoV-2 spike [38]. Thereby, the rapid emergence of several SARS-CoV-2 strains with increased number of mutations, particularly in the spike-encoding gene, that has been reported in some recent observations [13,20,21,22,23,24,25,26,27] (Table 1) questions whether an event (e.g., genetic or ecologic) has occurred that may have modified the virus replication process or the host-dependent virus selection to promote and spread variant viruses that are more transmissible than the previously-circulating strains and capable of escaping immune responses in some cases [30,32,39]. It raises fears of an over-amplification of the pandemic worldwide. Of particular concern are the mutations within the viral spike that attach the virion to the ACE2 cell-surface receptor [40] and serve as major target for neutralizing antibodies [36,41].

## 3. Mutations in the Spike Associated with a Risk of Neutralizing Immune Response Escape

Several mutations in the spike protein, including those in its receptor-binding domain (RBD; amino acids 333 to 527 of the spike) that binds to ACE2, increase the affinity of the trimeric form of this spike to ACE2 and thus increase viral replication or worsen viral cytopathic effects [42]. In addition, some mutations were reported to reduce the sensitivity to anti-SARS-CoV-2 antibodies [43,44]. The viral variants that we detected in July 2020 and named Marseille-4 (later classified as Nextstrain clade 20A.EU2) [9,12] harbor amino acid substitution S477N located in the spike RBD [9,12,45]. This substitution was reported to confer resistance to neutralization to multiple monoclonal antibodies [39]. Regarding the rapidly spreading SARS-CoV-2 variants from the United Kingdom (20I/501Y.V1) and South Africa (20H/501Y.V2), both harbor a substitution N501Y in the RBD that increases affinity to ACE2 [31,32] plus six additional substitutions including P681H for the 20I/501Y.V1 variant [30] or K417N and E484K for the 20H/501Y.V2 variant [31], which reduce the sensitivity to anti-SARS-CoV-2 antibodies [43,44]. Furthermore, these mutations can lead to immune escape, as shown in several in vitro studies [35,43,44]. Accordingly, beyond the uncertainty that remains about the duration of protective immune response against SARS-CoV-2 after a first episode of infection, a major concern comes from the appearance of amino acid changes in the spike of new variants. Genomic evidence of SARS-CoV-2 reinfections with 20I/501Y.V1, 20H/501Y.V2, and 20J/501Y.V3 variants were documented [46,47,48]. Regarding, for instance, the first of these reports, it involved a 78-year-old man with type-2 diabetes mellitus, diabetic nephropathy on hemodialysis, and chronic obstructive pulmonary disease in the UK [46]. This patient was diagnosed with SARS-CoV-2 during the first episode of the pandemic and exhibited mild illness. Then, 8 months later, he was reinfected with the 20I/501Y.V1 variant as documented by whole-genome sequencing, which caused a critical illness. In addition, we observed in our institute the case of several patients who had been infected in March–April 2020, then experienced clinical recovery and viral clearance as documented by qPCR negativity but were infected again during summer 2020 or later with a Marseille-4 variant that carries the S477N substitution in the spike [49]. Such clinical observation of reinfection with a Brazilian variant is corroborated by the observation that culturing in vitro, in the presence of neutralizing plasma, a SARS-CoV-2 isolate sensitive to highly-neutralizing plasma from a COVID-19 convalescent patient was associated with the occurrence of a deletion of amino acid F140 in the N-terminal domain (NTD, loop N3) of the spike after 45 days and of the E484K substitution after 73 days, followed by an insertion in the NTD, loop N5 [50]. Computational modeling was also reported to predict that this variant should escape neutralization antibodies [50]. Furthermore, such mutations could make the vaccine approach less effective or even harmful via antibody-dependent enhancement of viral uptake through binding to the immunoglobulin Fc receptor expressed on cells or complement receptor-bearing cells [51].

## 4. Intriguing High Error Rate in the New SARS-CoV-2 Variants

We can wonder about the mechanisms of the emergence and selection of these new highly transmissible SARS-CoV-2 variants. SARS-CoV-2, like other coronaviruses and RNA viruses, is evolving according to the quasi-species (mixtures of different viral populations) model characterized by continuous genetic variation as a result of a high error rate of RdRp [4,5]. Under positive selective pressure from the host, spontaneously generated mutations can be selected, leading to the emergence of variant viruses able to escape the host’s defense mechanisms [52]. These variants are strains that differ from all others by a set of several mutations and have reached a detectable population size. Among RNA viruses, coronaviruses appear to be quite complex, since they have the largest genome (almost 30,000 bases). They harbor two characteristics [53,54,55]: a RdRp combined with a proofreading activity conferred by the Nsp14 exoribonuclease and a homologous recombination mechanism associated with replication. High mutation rates and deleterious mutations should be prevented through the corrective action of Nsp14 that is able to remove nucleoside analogues after their incorrect insertion into the nascent RNA. Although the capacity of SARS-CoV-2 to evolve under the host’s immune pressure remains to be further characterized, this virus is likely to have a reduced tolerance to genetic drift in order to maintain the integrity of its large RNA genome, under the action of its Nsp14 exonuclease that counteracts the low RdRp fidelity [54]. As a result, the recent emergence of some variants with large sets of mutations is surprising and difficult to explain without evoking a particular induction and selective pressure. It can either result from a natural origin (such as an intra-host species evolution under the immune response pressure or a change in host species before reintroduction into humans) or be induced by a therapy (such as an antiviral therapy inducing escape variants, as previously reported decades ago for AIDS monotherapy [56]).

## 5. Selection of Highly Transmissible Variants: ‘Natural Hypothesis’

A first hypothesis for the emergence and selection of new SARS-CoV-2 variants, the natural hypothesis, is linked to the selective pressure of the host’s immune system. In this model, if the virus is exposed to the host’s immune response, including neutralizing and non-neutralizing antibodies, this can select for spike variants among the viral quasi-species [4]. One SARS-CoV-2 variant with substitution D614G in the spike, which increases the stability of S trimer complex and renders the virus more infectious, progressively increased in prevalence worldwide and was almost the only variant since the epidemic onset in Europe [56]. It corresponds to the selection of a new beneficial mutation that increases its frequency and fixes it in the population [57]. It has been, for instance, reported that functional SARS-CoV-2 spike variants with mutations in the RBD and NTD that confer resistance to monoclonal antibodies or convalescent plasma can be selected in vitro [58]. In another work, Li et al. studied the infectivity and reactivity to neutralizing antibodies and convalescent patients’ sera of 106 SARS-CoV-2 spike mutants including 26 with deletions at putative N-linked glycosylation sites, and they identified changes in infectiousness and sensitivity to neutralizing antibodies, including resistance in some cases [59]. In addition, Thomson et al., reported that the natural spike variant N439K can escape antibody-mediated immunity while maintaining fitness [60].

## 6. Selection of Highly Transmissible Variants: ‘Interventionist Therapy Hypothesis’

A second hypothesis to explain the emergence and selection of new SARS-CoV-2 variants, the interventionist therapy hypothesis, is linked to the selective pressure of treatments. In order to treat severe COVID-19, some patients received immunoglobulin-based immunotherapy with hyper-immune sera from convalescent COVID-19 patients [13,20,21,22,23,24,25,26,27,61] (Table 1). However, a study reported that, of the sera from 26 patients who recovered from COVID-19 and exhibited high titers of anti-SARS-CoV-2 immunoglobulins, only three effectively blocked the binding of the SARS-CoV-2 spike protein to ACE2 [62]. Studies by Greaney et al. and Liu et al. also reported that neutralization of spike harboring changes at some amino acid positions, including positions 484, 456, and 477, had decreased sensitivity to convalescent serum antibodies [35,39]. Recent in vitro results from Andreano et al. [50] also suggested that SARS-CoV-2 can evade suboptimal concentrations of neutralizing antibodies. These authors reported that after 45 days of culture (six subcultures) of wild-type SARS-CoV-2 with serial two-fold dilutions of neutralizing plasma, a variant with several spike mutations including the E484K substitution emerged and resisted high levels of neutralizing antibodies. Weisblum et al. also reported the selection of SARS-CoV-2 with mutations in the spike RBD by culturing in the presence of convalescent plasma [58]. In addition, it was reported that 15 patients with hematological malignancies, one patient with multiple sclerosis, and one patient with common variable immune deficiency, who had prolonged COVID-19 symptoms, improved their clinical symptoms and showed decreased SARS-CoV-2 RNA load between 7 and 14 days after receiving four units of COVID-19 convalescent plasma therapy (some of them having also received remdesivir) [63]. However, five of these patients remained positive for SARS-CoV-2 on nasopharyngeal swab, and unfortunately their viruses were not monitored by whole-genome sequencing, and information regarding the possible selection of variants is therefore missing. Additionally, the 20H/501Y.V2 and 20J/501Y.V3 variants were reported to escape from therapeutic antibodies and antibodies elicited by infection and vaccine immunization [64,65]. These data indicate that administration of sera from convalescent patients may favor the emergence of SARS-CoV-2 variants evading the immune response, as recently suggested [65].

Also worth noting are the cases of patients who received the antiviral nucleoside (adenosine) analog prodrug GS-5734, remdesivir, which sterically interacts with the viral Nsp12/RdRp to induce delayed chain termination [66]. The structure of the SARS-CoV-2 Nsp12/RdRp in complex form with Nsp7 and Nsp8, the template primer RNA, remdesivir, and Mg2+ ions was determined recently [67]. Only low-level resistance to remdesivir has been observed in vitro, in association with two amino acid substitutions (F480L and V557L) in the Nsp12/RdRp [68], while RdRp substitution D484Y has been observed in vivo in association with treatment failure [69]. This corroborates the observation that under subclinical concentrations of remdesivir, a variant of Ebola virus emerged with a single F548S substitution that confers fourfold to fivefold reduced susceptibility to remdesivir [70]. This mutation lies in the F-motif of the RdRp active site where mutations that confer remdesivir resistance occur in coronaviruses. It is worthy to note that RdRp substitution P323L (due to a substitution at nucleotide 14,408) was associated with an increased mutation rate [71]. Indeed, the median number of mutations within the viral genome was three in its presence versus one in its absence. A mechanism of action similar to that of remdesivir has been reported for the prodrug T-705 (favipiravir), a drug that is orally administered and reported to inhibit SARS-CoV-2 by lethal mutagenesis escaping the coronavirus repair machinery [72]. Similarly, the ribonucleoside analog NHC/EIDD-2801 antiviral activity is associated with increased viral mutation rates [73]. Altogether, these results suggest that nucleoside analog prodrugs may increase the mutation frequency of SARS-CoV-2, allowing the emergence of fast-spreading SARS-CoV-2 variants. Interestingly, remdesivir may also interact with and hinder the action of the Nsp14 exonuclease that has proofreading activity and excises mis-incorporated nucleotides [74]. Therefore, remdesivir could shut down the correcting activity and increase the mutation rate. A mutagenic effect was also predicted for remdesivir [75]. Remdesivir is an analog of adenine and is believed to compete with adenine triphosphate (ATP) during the viral replication. The mechanism of action of this molecule could lead to (i) RNA chain termination; (ii) non-obligate chain termination with modification of neighboring side chain; or (iii) delayed chain termination. Both non-obligate chain termination and delayed chain termination have been proposed for remdesivir [66,67,76]. In addition, it was reported that tautomers (structural isomers that differ from one another regarding the position of protons and double bonds) of RNA bases could play a crucial role in mutagenesis [77]. Adenine has the ability to adopt amino and imino tautomeric forms involving the exocyclic group at the 6-position. In Jena’s article [75], the role of different tautomers in their base-pairing abilities was studied to further understand the role of remdesivir in the generation of mutations. It was found that remdesivir can adopt both amino and imino tautomeric conformations to base-pair with RNA bases. While the insertions of G and U appeared as preferred pairs against the amino tautomers of this drug, the insertion of C is mainly possible against the imino tautomers. The author concluded that both amino-remdesivir: G and imino-remdesivir: C pairs could be quite mutagenic. An experimental work by Szemiel et al. [78] recently demonstrated how serial in vitro passages of SARS-CoV-2Engl2 in cell culture media supplemented with remdesivir selected for drug-resistant viral populations. They found that remdesivir triggers the selection of SARS-CoV-2 variant with a E802D mutation in the RdRp sufficient to confer decreased sensitivity to remdesivir without affecting viral fitness. Another mutation, I168T, was observed in the Nsp6. The analysis of more than 200,000 sequences also revealed the occurrence of 22 mutations in the spike, including changes in amino acids E484 and N501 corresponding to mutations identified in emerging SARS-CoV-2 variants in the UK (20I/501Y.V1) and in South Africa (20H/501Y.V2). These results clearly indicate that E484K and N501Y mutations can arise in vitro in the absence of immune selection under the sole selection pressure of remdesivir.

## 7. Could Remdesivir and/or Convalescent Plasma Experimental Therapy Promote the Emergence of Highly Transmissible SARS-CoV-2 Variants?

**Figure 2:**
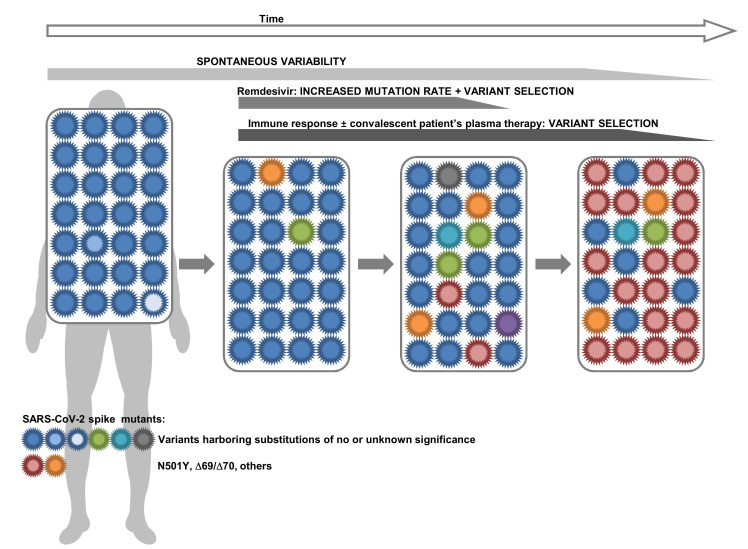
Possible mechanism of occurrence and selection of SARS-CoV-2 spike variants.

The above results led to the question of whether the N501Y-harboring SARS-CoV-2 variants may have arisen due to remdesivir administration and concurrent or subsequent selection by the host’s immune response and/or the administration of antibodies from convalescent patients’ sera (Figure 2). Indeed, delayed viral clearance and remdesivir and/or convalescent plasma could together promote the emergence of new SARS-CoV-2 variants. As shown in Figure 2, spontaneous viral genetic variability generates SARS-CoV-2 mutants that can be selected by the immune response but also by convalescent plasma therapy or remdesivir. The longer the viral replication lasts, the greater the probability of generation of SARS-CoV-2 mutants and subsequent selection is. Such phenomenon has been well demonstrated for instance for HIV, with the spontaneous generation of viral mutants during uncontrolled viral replication then selection by antiretroviral therapy of those mutants that are less susceptible to the administered drugs [14,79]. In addition, remdesivir could increase the mutation rate [69,74,75,76].

As a matter of fact, several cases have been reported for which the emergence of SARS-CoV-2 variants with mutations within the viral spike was evidenced in immunocompromised patients with prolonged SARS-CoV-2 infection who had received remdesivir and/or convalescent plasma or anti-spike antibodies (Table 1). The mutation rate was in some cases dramatically greater than that estimated for SARS-CoV-2 (9.8 × 10^−4^ substitutions/site/year, or 29.3 substitutions/genome/year [80]). The first case occurred in a 45-year-old man with a severe antiphospholipid syndrome who was recurrently diagnosed with SARS-CoV-2 over approximately 5 months in Boston, USA [13]. He was treated with glucocorticoids, cyclophosphamide, and eculizimab. This patient received four 5-day or 10-day courses of remdesivir around days 0, 72, 105, and 151 after first viral detection. He also received an antibody cocktail targeting the SARS-CoV-2 spike. Sequencing of sequential samples showed that amino acid changes had occurred within the spike and its RBD in 57% and 38% of cases, these regions being over-mutated since they represent only 13% and 2% of the viral genome, respectively. These changes included substitutions P9L and Q183H and deletion delta142/144 in the NTD; substitutions I870V and A1020S in the C-terminal domain; as well as substitutions N440D, T478K, E484K/A, F486I, Y489H, Q493K, S494P, and N501Y in the RBD. Strikingly, this latter change that was found from day 128 until day 152 is the one harbored by SARS-CoV-2 variants that have expanded since October, mostly in the United Kingdom [31]. One non-synonymous mutation occurred in the envelope and three in the nucleocapsid. A single synonymous mutation was observed in the RdRp. SARS-CoV-2 in respiratory samples was found to be infectious when tested at days 75 and 143. A second case was in a hypogammaglobulinemia patient who was treated with B cell depletion and who was followed over 101 days with viral genome sequencing from 23 sequential respiratory samples [20]. This patient received two courses of remdesivir at days 41 and 54 and SARS-CoV-2 convalescent patients’ plasma at days 63, 65, and 93. Low-frequency variant analysis showed the occurrence of the spike variant as soon as on day 45, then of the N501Y variant at 33% frequency on day 55 that was no longer detected on day 66 when variants with changes in Nsp2 and RdRp (V157L) occurred. This latter variant was later replaced after convalescent plasma administration by variants that differed according to the time point and harbored spike substitutions Y200H, T240I, P330S, D796H, and the Delta69/70 double deletion found in the 20I/501Y.V1 UK variant. The Delta69/70 single mutant had two-fold higher infectivity compared to the wild type. These findings could possibly explain why such variants harboring Delta69/70 deletion and N501Y substitution emerged in countries such as the UK where remdesivir is routinely used for the treatment of hospitalized SARS-CoV-2 patients with chronic infection. Seven other studies reported cases of 50–75-year-old immunocompromised patients with hematological malignancies or severe hypogammaglobulinemia who experienced viral shedding between 41 and 268 days and received one to four cures of remdesivir and/or one or five administrations of convalescent plasma [21,22,23,24,25,26,27]. Four patients eventually died at days 74–271 after SARS-CoV-2 diagnosis. Viral genomes showed in three patients who received remdesivir the occurrence of 6 to 21 amino acid changes. These included the deletion of amino acid Y144, and substitutions H69Y/P, V70G, D215G, E484G/K, and N501T, all these changes having occurred at spike positions that also harbor mutations in variants 20I/501Y.V1, 20H/5017.V2, or 20J/501Y.V3. Finally, we have been able to observe in our series that, of 212 severely immunocompromised COVID-19 patients hospitalized that notably included 22 patients with lymphoma, only two had viral persistence beyond 70 days. One had received a course of remdesivir and a convalescent plasma outside our institute, and his virus accumulated mutations including two in the spike (Table 1).

## 8. Conclusions

We therefore hypothesize that both remdesivir and convalescent plasma therapy, alone and in combination, play a role in the generation and selection of amino acid changes in the SARS-CoV-2 spike protein. This means that in studies that report cases of viral persistence in immunocompromised patients, it is essential to specify the therapy that has been used and to sequence genomes of persistent viruses. It is indeed essential in the context of this disease, as in other viral diseases, to keep in mind that the antivirals given in monotherapy to people who are chronic viral carriers, as has been the case for HIV or hepatitis C, easily select resistant viral mutants. It is urgent to explore why new SARS-CoV-2 variants with spike mutations suddenly arose in France, the UK, South Africa, or Brazil. One hypothesis is a genetic change due to an ecological accident such as transmission from humans to another species and then reinfection of humans, as it is suspected with minks [9,10,81]. Another hypothesis that could shed light on these aberrant events involves chronically ill patients treated with experimental therapies at concentrations that did not allow eliminating the virus and favored the emergence of variants from the residual viral load. Some immunocompromised patients with persistent SARS-CoV-2 infection [13,20] were administered remdesivir, which possibly increases the frequency of mutations, and received convalescent plasma or anti-spike antibodies that were reported to drive the occurrence of spike escape mutations. New SARS-CoV-2 mutants or variants that emerge in immunocompromised patients with prolonged viral shedding could secondarily spread to non-immunocompromised patients to an extent that depends on the epidemiological and clinical characteristics of these viruses. While waiting to learn more, it is essential to use experimental therapies with extreme caution and to monitor the possible emergence of SARS-CoV-2 variants in these patients. This means that in studies that reported cases of viral persistence in immunocompromised patients, it is essential to specify the therapy that has been used, which lacked for some patients [82], and to sequence genomes of persistent viruses, which lacked in a convalescent plasma therapy study [63]. The characterization of variants relies on whole-genome sequencing that may be substantially under-reported. An evolution of clinical practices is required, and the promoters of immunotherapy and antiviral therapy trials in COVID-19 patients should imperatively organize the monitoring of SARS-CoV-2 variants by whole-genome sequencing, in particular for patients who are immunosuppressed.

## Figures and Tables

**Table 1:** Epidemiological, virological, and clinical features of cases of prolonged SARS-CoV-2 infections in immunocompromised patients who received remdesivir.

Reference	Gender, age (years)	Cause of immunodepression	Duration of viral shedding (days) ^a^	Treatment				Number of amino acid substitutions/deletions (including in spike (S) gene)	Outcome
				Remdesivir	Convalescent plasma	Anti-spike antibody cocktail	Other(s)		
[13]	Male, 45	Severe antiphospholipid syndrome	151	Day 0 (5 days), day 72 (10 days), day 105 (5 days), day 151 (2 days)	-	Day 143	Cyclophosphamide, eculizumab, rituximab, ruxolitinib, prednisone, hydroxychloroquine, antiviral antibody cocktail	45 mutations (24 non-synonymous), 34 deletions. 11 mutations and 9 nucleotide deletions occurred between days 18–25 and days 75–81, 10 mutations and a 1-nucleotide deletion occurred between days 75–81 and days 128–130, 11 mutations and 24 nucleotide deletions occurred between days 128–130 and 143–152; spike: 12 non-synonymous substitutions, among which substitution N501Y present in variants 20I/501Y.V1, 20H/501Y.V2 and 20J/501Y.V3 ^b^, E484K present in variants 20H/501Y.V2 and 20J/501Y.V3 ^b^, and deletion Y144- present in variant 20I/501Y.V1	Death on day 154
[20]	N.a.	Marginal B cell lymphoma (received B cell depletion therapy; hypo-gammaglobulinemia)	101	Day 41, day 54, day 93	Days 63, 65, 95	-	-	41 nucleotide substitutions and 6 nucleotide deletions. 1 nucleotide substitutions after 1st RDV course, 15 after 2nd RDV course and 1st and 2nd CP administrations, 15 after 3rd RDV course, 10 after 3rd CP administration. Spike: 10 nucleotide substitutions and 6 nucleotide deletions among which substitution N501Y and deletion H69/V70 present in 20I/501Y.V1 ^b^	Not reported
[21]	Male, 60	Mantle cell lymphoma	156	Days 30, 122	Days 33, 122	-	CD20 bispecific antibody, second B-cell directed antibody, cyclophosphamide, doxorubicine, prednisone	9 mutations, 6 non-synonymous in ORF1a (n = 4), ORF1b (n = 1), ORF3a (n = 1). 2 mutations occurred at day 29, 4 at day 93, 3 at day 106 (last sequencing)	Pursued home hospice care
[22]	Male, 75	Multiple myeloma	71	Days 5–10	Days 2, 58	-	Dexamathasone (days 63–74)	9 amino acid substitutions and 12 amino acid deletions in the spike protein between days 4 and 67 including 2 and 7 substitutions at days 13 and 67, respectively, and 11 and 1 amino acid deletions at days 67 and 72, respectively. Among these amino acid changes are D215G present in 20H/501Y.V2 ^b^, Y144 deletion present in 20I/501Y.V1 ^b^, LAL242-244 deletion present in 20H/501Y.V2 ^b^, and N501T at a position mutated in variants 20I/501Y.V1, 20H/501Y.V2 and 20J/501Y.V3 ^b^	Death on day 74
[23]	Male, 60–70	Non-Hodgkin lymphoma	268	Days 47–51, days 77–86, days 178–182, days 205–209	Day 88	-	Darunavir/ritonavir, hydroxychlorquine, IV methylprednisolone, tocilizumab, ceftaroline	13 amino acid substitutions between days 34 and 238 (15 in ORF1a, 1 in ORF1b, 6 in spike, 3 in ORF3a; 6, 2 and 5 at days 54, 76 and 238, respectively). Spike: 6 substitutions among which H69Y/P and V70G at positions mutated in variant 20I/501Y.V1 ^b^	Death on day 271
[24]	Male, 50–60	Organ transplantation	145	Days 140–150	-		Tacrolimus, mycophenolate mofetil, prednisone, ivermectine (days 56–60)	At day 105: 16 substitutions (7 in ORF1ab, 4 in spike, 1 in ORF3a, 1 in ORF7b, 3 in nucleocapsid), 3 deletions. Spike: 4 substitutions S13I, T95I, E484G, F490L, and 3 deletions 141–144, 244–247, 680–687 (E484G at a position mutated in variants 20H/501Y.V2 and 20J/501Y.V3; Y144 deletion is present in 20I/501Y.V1 ^b^)	
[25]	Female, 70s	Follicular lymphoma	>134	-	Five times between days 45 and 115	-	Steroids	44 mutations including 28 amino acid substitutions and 12 nucleotide deletions. 4, 1, 9, 3, 2, 2, 2, and 8 mutations occurred at days 7, 13, 21, 30, 34, 42, 63, 77, and 134, respectively. Spike: 5 mutations of which 3 amino acid substitutions, including E484K present in variants 20H/501Y.V2 and 20J/501Y.V3 ^b^; one inframe deletion Y144 present in variant 20I/501Y.V1 ^c^	Death on day 156
[26]	Male, early 50s	Kidney-transplant recipient	41	-	Day 1	-	Tacrolimus, mycophenolate mofetil, prednisone, tocilizumab (day 2)	4 mutations at days 21–27 including 3 substitutions (1 in ORF1a and 2 in spike: E484K and Q493R/K), and 2 deletions (spike amino acids 141–144 and 243 244). E484K is present in variants 20H/501Y.V2 and 20J/501Y.V3 ^b^; deletion Y144- is present in variant 20I/501Y.V1 ^b^; deletion LA243–244 is present in variant 20H/501Y.V2 ^b^	Death on day 94
[27]	Female, 71	Chronic lymphocytic leukemia, acquired hypogammaglobulinemia	105	-	Days 70, 80	-	-	6 nucleotide substitutions at day 49 including 5 amino acid substitutions (2 in the spike), and 1 deletion. 3 additional amino acid substitutions (2 at day 70, 1 at day 85) and 1 additional deletion at day 70 (a 12-bp deletion in the spike N-terminal domain of S1 region)	N.a.
Present case	Male, 66	Burkitt lymphoma	112	Day 40 (6 days)	Days 77, 78	-	Hydroxychloroquine 10 days, azithromycin 5 days, dexamethasone 10 days	15 mutations including 2 amino acid substitutions in the spike: L18F and R682Q. 1, 2, 2, 4, 3, 1 and 1 occurred at days 14, 29, 47, 64, 78, 84, and 101, respectively	Death on day 119

^a^ As assessed by qPCR; ^b^ 20I/501Y.V1 = “UK” variant, 20H/501Y.V2 = “South African” variant, and 20J/501Y.V3 = “Brazilian” variant; ^c^ at the end of second cure of remdesivir. CP, convalescent plasma; N.a., not available; RDV, remdesivir.

## Data Availability

Data are available from the corresponding author upon reasonable request.
